# Coherent response of the Indian Monsoon Rainfall to Atlantic Multi-decadal Variability over the last 2000 years

**DOI:** 10.1038/s41598-020-58265-3

**Published:** 2020-01-28

**Authors:** Pothuri Divakar Naidu, Raja Ganeshram, Massimo A. Bollasina, Champoungam Panmei, Dirk Nürnberg, Jonathan F. Donges

**Affiliations:** 1 CSIR-National Institute of Oceanography, Dona Paula, 403004 Goa, India; 2 School of Geosciences, University of Edinburgh, Edinburgh, UK; 3 Academy of Scientific and Innovative Research (AcSIR), CSIR-NIO, Goa, India; 4 GEOMAR, Wischhofstrasse 1-3, 24148 Kiel, Germany; 5 Postdam Institute for Climate Impact Research, P.O. Box 601203, D-14412 Postdam, Germany; 6 Planetary Boundary Research Lab, Stockholm Resilience Center, Stockholm University, Stockholm, Sweden

**Keywords:** Palaeoceanography, Physical oceanography

## Abstract

Indian Summer Monsoon (ISM) rainfall has a direct effect on the livelihoods of two billion people in the Indian-subcontinent. Yet, our understanding of the drivers of multi-decadal variability of the ISM is far from being complete. In this context, large-scale forcing of ISM rainfall variability with multi-decadal resolution over the last two millennia is investigated using new records of sea surface salinity (δ^18^Ow) and sea surface temperatures (SSTs) from the Bay of Bengal (BoB). Higher δ^18^Ow values during the Dark Age Cold Period (1550 to 1250 years BP) and the Little Ice Age (700 to 200 years BP) are suggestive of reduced ISM rainfall, whereas lower δ^18^Ow values during the Medieval Warm Period (1200 to 800 years BP) and the major portion of the Roman Warm Period (1950 to 1550 years BP) indicate a wetter ISM. This variability in ISM rainfall appears to be modulated by the Atlantic Multi-decadal Oscillation (AMO) via changes in large-scale thermal contrast between the Asian land mass and the Indian Ocean, a relationship that is also identifiable in the observational data of the last century. Therefore, we suggest that inter-hemispheric scale interactions between such extra tropical forcing mechanisms and global warming are likely to be influential in determining future trends in ISM rainfall.

## Introduction

The Indian summer monsoon (ISM) is a large-scale coupled land-ocean-atmosphere phenomenon that plays a dominant role in transporting water vapour to the Indian subcontinent^1^. Despite the strong bearing and societal relevance of the monsoon, reliable projections of ISM rainfall remain a challenge^2^, mainly because of the complex dynamics and the myriad of interactions with other tropical and extra-tropical processes across various timescales^3^.

On a year to year basis, the ISM exhibits considerable complex and time-varying interactions with the El Niño/Southern Oscillation (ENSO)^4^, the Indian Ocean Dipole^5^ and Tibetan snow cover^6^. Additionally, intraseasonal active or break events on short timescales, presumably associated with internal dynamics of the monsoon system itself, have large impacts on the interannual variability of monsoon rainfall^7,8^. Example includes the famous break of July 2002, where rainfall was reduced by 50%^9^. These large year-to-year variations in rainfall associated with internal and external interactions pose a considerable challenge for Indian monsoon forecasting^10^ and for attributing monsoon variability to large-scale forcings. This is particularly true for multi-decadal and longer time scales given that observational data only covers few cycles and paleoclimate records span longer time scales which fill this gap.

There is currently a considerable debate on the impact of different forcing agents versus the role of internal variability in determining past and future long-term trends of the ISM^11^. Global climate model experiments tend to suggest an intensification of the ISM in response to global warming partly because of the larger water-holding capacity of a warmer atmosphere, resulting in an estimated 10% increase by the end of the 21^st^ century^12^; yet, there are large inter-model discrepancies (e.g., ref. ^10^). This is in striking contrast with the recent observational record, which indicates an overall decrease of the ISM rainfall during the period 1950–2012^13–15^, followed by a weak revival between 2012–2014^16^. Decadal-scale internal climate variability associated with ENSO and the Atlantic Multi-decadal Oscillation (AMO) can also play an important role in explaining the observed variations in NH monsoon precipitation during the last century^17^ and possibly beyond^18,19^. Thus a number of drivers, can contribute to modulating ISM rainfall, including internal variability of the climate system as well as interactions with natural and anthropogenic factors, either of global (e.g., greenhouse gases) or regional (e.g., anthropogenic aerosols and land-use) nature^11,20–22^.

More integrated studies of the wider Northern Hemisphere (NH) summer monsoon suggest coherent changes of monsoon precipitation and circulation at hemispheric scales^23^. The relatively coherent response of the various monsoons organised into the NH monsoon system to natural and anthropogenic forcing suggests that monsoon rainfall is sensitive to common hemispheric and/or planetary scale controls. Implicit in this view is the hypothesis that large-scale forcings may override regional-scale factors both natural and anthropogenic in determining future NH monsoon rainfall trends^23,24^. Conversely, several studies also suggest a varying response of the individual monsoon systems to the same forcing due to internal feedback dynamics^25^. Therefore, a better understanding of the large-scale controls of past decadal-scale variability of the monsoon is key to achieve more robust projections of its future changes. However, observations are spatially and temporally limited, mostly to the 20^th^ century, which only allow identifying few long-term fluctuations^17^. Further extending these records back in time using paleoclimate reconstructions is essential to reduce current uncertainties, to identify its local and global scale forcing mechanisms, and to quantify the background monsoon variability on top of which shorter timescale fluctuations as well as longer-term anthropogenic-induced changes are superimposed^24^. Here we present a new record of ISM rainfall and SST variability from the Northern Indian Ocean spanning the last 2,000 years at multi-decadal resolution and investigate the large-scale forcing mechanisms of ISM rainfall.

## Salinity Variations in the Bay of Bengal

**Figure 1:**
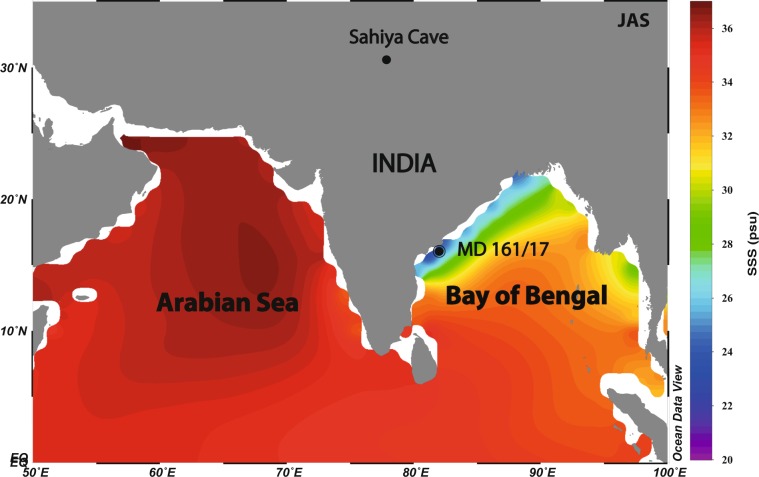
Location of MD161/17 Core and July through September salinity pattern in the northern Indian Ocean. Note MD161/17 Core was retrieved from the Krishna-Godavari Basin where a distinct low salinity prevails due the heavy over head precipitation and river discharge of Krishna, Godavari Rivers during Indian summer monsoon.

The Bay of Bengal (hereafter BoB; see Fig. 1) is a natural laboratory for reconstructing past ISM rainfall variations for two main reasons: Firstly, the BoB receives an estimated annual freshwater discharge of 2950 km^3^ from major (Ganges-Bramhaputra and Irrawaddy) and minor (Mahanadi, Krishna and Godavari) rivers fed by the ISM monsoon precipitation and glacier melt water^25^. Thus the catchment of these rivers cover a large swath of the summer monsoon rainfall regions of North, Northeast and Central India. Secondly, the BoB directly receives heavy precipitation particularly in the northern parts. Precipitation plus runoff exceeds evaporation on an annual cycle. Fresh water from these sources is mixed into the upper layers of the BoB interacting with the surface circulation. After the summer monsoon some of the strongest lowering of seasonal salinity occurs along the Coromandel Coast in the northern and western BoB (Fig. 1). We exploited this prominent low salinity cap to reconstruct ISM rainfall and SST records from the Sediment Core MD161/17. The core site receives sediments from Mahanadi, Krishna and Godavari, and its tributaries, draining the northern portion of the Indian plateau, leading to enhanced sedimentation and expanded sediment sequence in the Krishna-Godavari Basin^26,27^.

## Proxy Records of Indian Monsoon Rainfall

In this study, we use Mg/Ca (SST) and δ^18^O calcite (δ^18^Oc) from the calcite shells of planktonic foraminifera *Globigerinoides ruber* (hereafter *G. ruber*) to derive δ^18^Owater (δ^18^Ow) to reconstruct ISM rainfall variability in the BoB at muti-decadal to centennial time scales (see suppl. material for further details). δ^18^Ow exploits the higher O^16^/O^18^ isotopic ratios of precipitation associated with rainfall as opposed to sea water which has lower O^16^/O^18^ ratios after appropriate corrections. On land, δ^18^O of precipitation exhibits spatial variability and shows a negative relationship with precipitation which could vary over the monsoon season^24^. However, the effects of such spatial and temporal variability are minimised in our study as the BoB receives rainfall from a large area of the Indian monsoon fed region and salinity anomalies persist over the monsoon and post monsoon periods. Also, the δ^18^Ow and salinity show a strong positive relationship (r^2^ = 0.89 with a slope of 0.15) in the BoB^28^. Therefore, δ^18^Ow, at least in a qualitative sense, reflects salinity variations related to monsoon rainfall at the core site. Calcite tests of *G. ruber* are used for reconstructions because *G. ruber* fluxes from sediment trap time series from the BoB suggest that they record average surface conditions in the BoB^29^. Previous studies have successfully used *G. ruber* to reconstruct ISM rainfall variability at millennial scale in the BoB since the last glacial period^30,31^. High sedimentation rates (0.2 to 0.6 cmyr^−1^) in Core MD161/17 allow us to reconstruct ISM rainfall variability at multi-decadal temporal resolution for the first time with robust chronology derived from 11 radiocarbon dates of mixed planktonic foraminifera species (Fig. S1).

## Results

**Figure 2:**
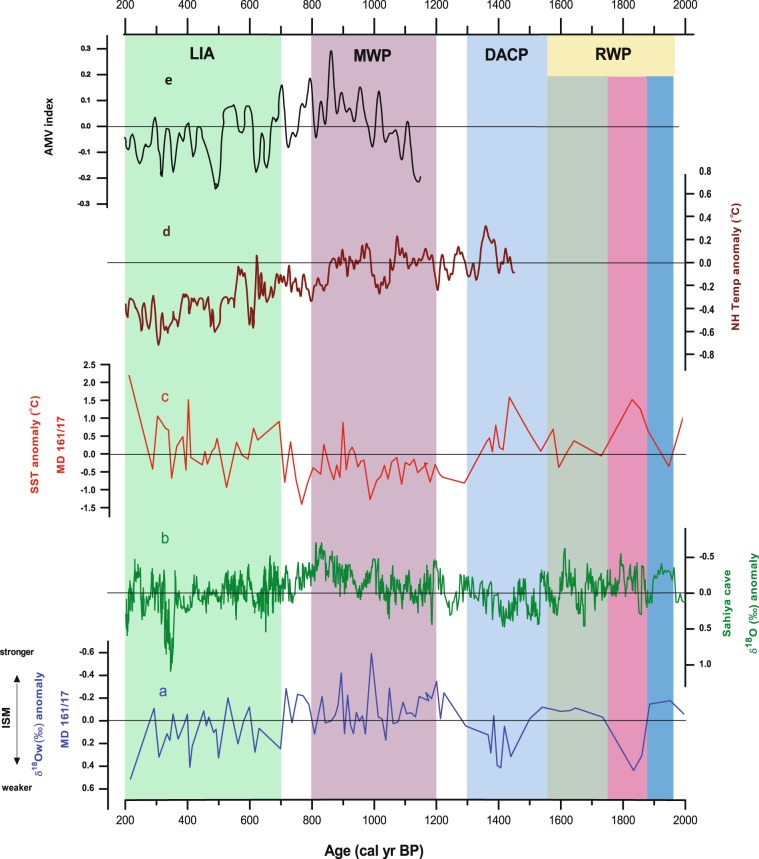
Variability of Indian summer monsoon rainfall over last two millennia; (**a**) δ^18^Ow anomaly derived from δ^18^Ow from δ^18^Oc of Globigerinoides ruber from MD161/17 Core; (**b**) δ^18^Ow anomaly of Sahiya Cave obtained from the δ^18^O of speleothem^19^; (**c**) sea surface temperature anomaly from MD161/17 core obatined from the Mg/Ca ratios in Globigerinoides ruber; (**d**) Northern Hemisphere Air Temperature anomaly^32^, (**e**) Multi-decadal variability of Atlantic multi-decadal oscillations^51^.

The δ^18^Ow record shows a large variability (1.1‰) over the last 2000 years, with high and low δ^18^Ow values representing decrease and increase of ISM rainfall respectively (Fig. S2). Figure 2a presents δ^18^Ow as positive and negative anomalies over the mean of the record. Major portions of the Roman Warm Period (1950 to 1550 years before present (BP); RWP) are associated with negative δ^18^Ow anomalies, suggesting higher ISM rainfall. During Dark Age Cold Period (DACP) (1550 to 1300 year BP), high positive δ^18^Ow anomalies are indicative of weaker ISM rainfall. The Medieval Warm Period (MWP) from 1200 to 800 years BP is characterized by negative δ^18^Ow anomalies compared to the preceding period (i.e. 2000 to 1300 years BP), which reveals that BoB experienced highest ISM rainfall and strong freshening during this MWP over the last 2000 years. In contrast, during the Little Ice Age (LIA), positive δ^18^Ow anomalies indicate a decrease in ISM rainfall from 700 to 200 years BP. Our ISM rainfall record from the BoB shows similarity with the speleothem record from the Sahiya Cave (Fig. 2b) with statistically significant positive correlation at zero-lag (Fig. S3) reflecting continental rainfall over the Northern Himalayan foothills^19^ (Fig. 1). This confirms that our reconstructed variability in ISM rainfall is broadly representative of a large swath of Northern & Central India including the Indo-Gangetic plains.

**Figure 3:**
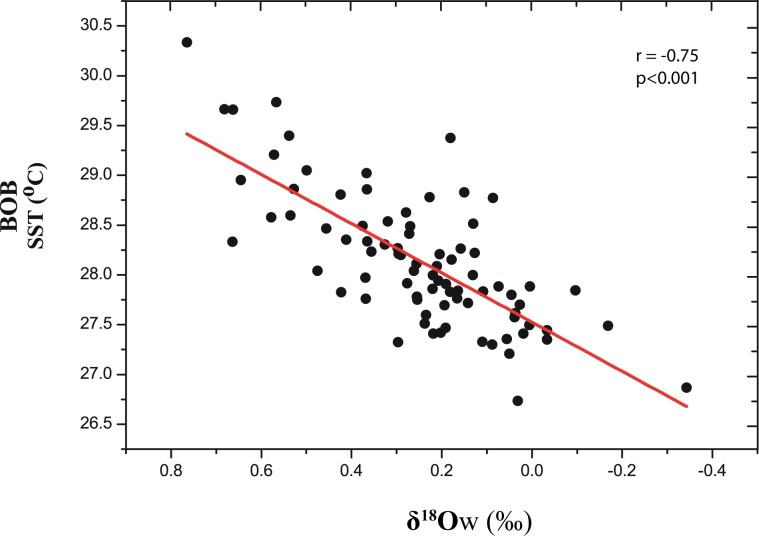
Relationship between the sea surface temperature and Indian Summer Monsoon Rainfall varibility over last two millennia. Here the depleted and enriched δ^18^Ow represent more and less ISM variability respectively. Note the significant negative correlation between the SST and ISM.

Reconstructed SST from foraminiferal Mg/Ca ratios over the last 2000 years varies between 26.7°–30.3 °C (Fig. S2). SST anomalies are more than 1 °C warmer during LIA and DACP and about 1 °C cooler during the MWP as compared to the mean SST (Fig. 2c). Figure 3 shows the strong negative correlation between reconstructed ISM rainfall (represented by δ^18^Ow) and Mg/Ca based SST (r = −0.75; p < 0.001; n = 84). Cross correlation analyses of these two time series records also showed a significant negative correlation with zero-lag suggesting a strong temporal coupling between BoB SST and ISM rainfall over the last 2000 years at this core location (Fig. S4 and Table S2). In Fig. 2d, we compare the anomalies of ISM rainfall and BoB SST with the Northern Hemisphere surface air temperature (NHT) anomaly data of Mann and Jones^32^ for the last 1500 years. The composite NHT is derived from Atlantic and European regions but also include data from central and eastern Eurasian regions^30^. Increased ISM rainfall and cooler BoB SSTs occur during periods of warmer NHT and vice versa (Fig. 2d). Also increased and decreased ISM rainfall during MWP and LIA corresponds with increase and decrease of AMO respectively (Fig. 2e). Kernel cross-correlation (red line) analysis of δ^18^Ow anomaly (proxy of ISM rainfall) and NHT show significant positive correlation at zero year lag (Fig. S5). In summary, our reconstruction reveals the positive temporal relationship between ISM rainfall and Eurasian temperature, as well as the inverse relationship between ISM rainfall and BoB SST. In the following sections, we further explore these links by using recent (last 100 years) observations which, despite being temporally limited, provides extensive spatial coverage useful to understand the nature and mechanisms underpinning decadal ISM rainfall variability in our reconstructions of the last 2000 years.

## Discussion

The Indian summer monsoon is a fully coupled ocean–land–atmosphere phenomenon whose fundamental driving mechanism can be identified in the differential heating between the land and the ocean to the south. During boreal spring, the land region over Asia and SE Asia warms faster than the ocean due to the lower heat capacity, setting up a low sea level pressure anomaly over Northern India and the Middle East. The resulting southward pressure gradient drives a cross-equatorial flow at the surface and a return flow aloft, forming a thermally-direct meridional cell^8^. A number of studies has found the Himalayas and Tibetan Plateau to also play an important thermal and mechanical role contributing to anchoring and intensifying the monsoon circulation by, respectively, heating the mid troposphere as well as by preventing cold air of mid-latitude origin to be advected to the south^33,34^. The large-scale inter-hemispheric thermal contrast that develops in late-spring and early summer leads to the northward seasonal migration of the Inter-Tropical Convergence Zone^10^. A large proportion of monsoon rainfall falls over northeastern peninsular India and in other orographic hotspots such as the Western Ghats and Arakan Range of Myanmar^35^. Once the monsoon is established over the Indian subcontinent, increased (land and ocean) evaporation and cloud cover lead to summertime cooling of the Indian Peninsula as well as of the north-equatorial Indian Ocean, with additional contribution from increased upwelling driven by the intense monsoon winds over its western sector^1^. As the Himalayas acts as a physical barrier for the southerly moisture-laden flow, precipitation and associated surface cooling are confined largely to the Indian Subcontinent, while the Tibetan Plateau is mainly dry and warm^35^. This maintains the meridional temperature gradient sustaining monsoon circulation and rainfall during the summer monsoon season^36^. Our reconstructions shown in Fig. 2a,c capture two key characteristics of the fundamental mechanics underpinning the annual cycle of the ISM^1^: salinity variations in the BoB reflecting monsoon rainfall and freshwater flux and^2^ lowering of the surface SSTs of the northern Indian Ocean during the monsoon period representing evaporative cooling associated with intensified monsoon winds and cloud cover. The inverse relationship (Fig. 3) and the coherent response (Fig. 2a,c) of these two reconstructed elements provide assurance that the changes we see are related to ISM variability. Furthermore, the close correspondence between these reconstructions and the NHT records (Fig. 2d,e) support the causal link between NH temperature and ISM rainfall via seasonal changes in the meridional surface/tropospheric thermal gradients^23,24^.

## Drivers of Multi-decadal ISM Rainfall Variability

**Figure 4:**
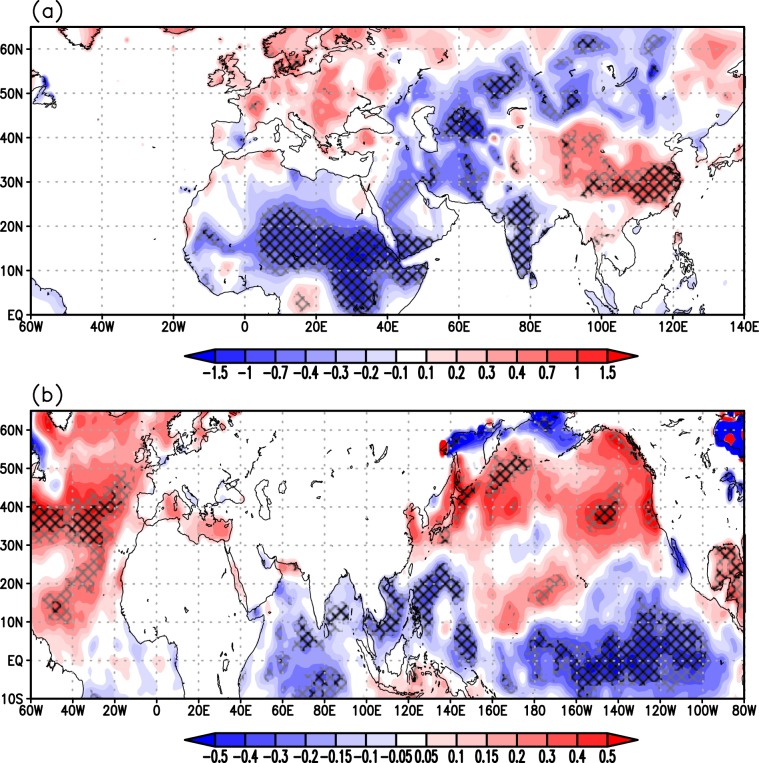
Regressions of observed low-pass filtered June-September (JJAS) mean (**a**) surface temperature (°C) and (**b**) sea-surface temperature (°C) on observed JJAS All-India summer monsoon rainfall during 1901-2012. All data were linearly detrended prior to the analysis. The analysis focuses on decadal or longer scale variability, isolated by applying a low-pass Lanczos filter with a cut-off frequency of 10 years to JJAS-mean data. The cross-hatching marks regions where the correlation exceeds the 90% (grey) and 95% (black) confidence levels, estimated by using a Monte Carlo approach with 1,000 random samples of the time series^68^. Land temperature data are from the Climate Research Unit (CRU), University of East Anglia, United Kingdom, dataset (CRU-TS3.21) at 0.5° × 0.5° resolution^69^, while sea surface temperature are from the Hadley Centre Sea Ice and Sea Surface Temperature data set (HadISST) dataset version 1 at 0.5° × 0.5° resolution^70^. Rainfall data are taken from the homogeneous rainfall data set of 306 raingauges in India, developed by the Indian Institute of Tropical Meteorology^71^.

We further explore the link between large-scale temperature changes and ISM variability at multi-decadal scale by means of observational data during 1901–2012. To make a direct comparison with the multi-decadal proxy records we remove interannual and short time scale variability in the observational data by low-pass filtering. This isolates the decadal or longer time-scale components of the signal from higher-frequency variability which is known to be largely influenced by internal monsoon dynamics^10^. The purpose of using observational data here is twofold^1^: to evaluate whether seasonal features such as cooling of the BoB related to monsoon circulation are represented in decadal time scales and^2^ to identify large-scale forcing mechanisms of All-India Monsoon Rainfall (AIMR) fluctuations at these time scales. Figure 4 presents the spatial map of June-September (JJAS) surface temperature anomalies associated with decadal or longer variations in the AIMR. At the surface, excess AIMR is associated with significant positive temperature anomalies over Eurasia and in particular eastern Asia and the Tibetan Plateau (Fig. 4a) and negative temperature anomalies over the Indian Subcontinent and the Tropical Indian Ocean (Fig. 4b). The elevated heating over the Tibetan Plateau, concurrently with slight cooling of the north equatorial Indian Ocean, has been identified as a dominant factor driving the annual cycle and interannual variability of the monsoon system^7,37^. Further afield, a strong positive association between ISM rainfall and SST anomalies in the North Atlantic Ocean and surface temperature over western Eurasia is evident in Fig. 4b ^38^. Large basin-wide sea surface temperature anomalies are also identifiable across the Pacific, with cooling along the equatorial central-eastern Pacific, and an extensive warming in the extra tropics stretching from Japan to the western coast of the US. The tropical anomalies are somewhat reminiscent of a La Niña-like cooling in the eastern equatorial Pacific. However, the pattern also features substantial differences in two important and fundamental characteristics of the actual La Niña sea surface temperatures anomalies or of its longer-lived manifestation, the Pacific Decadal Oscillation (PDO)^39^. First, it does not display zonal opposite-sign SST anomalies over the western equatorial Pacific, and second, the anomalies do not extend over the northeastern Pacific. We will return to this point later. Overall, this corroborates the assembled paleo-reconstructions presented in Fig. 2 and allows us to place ISM rainfall variability over the last 2000 years in the wider context of mechanisms associated with NH climate variability.

**Figure 5:**
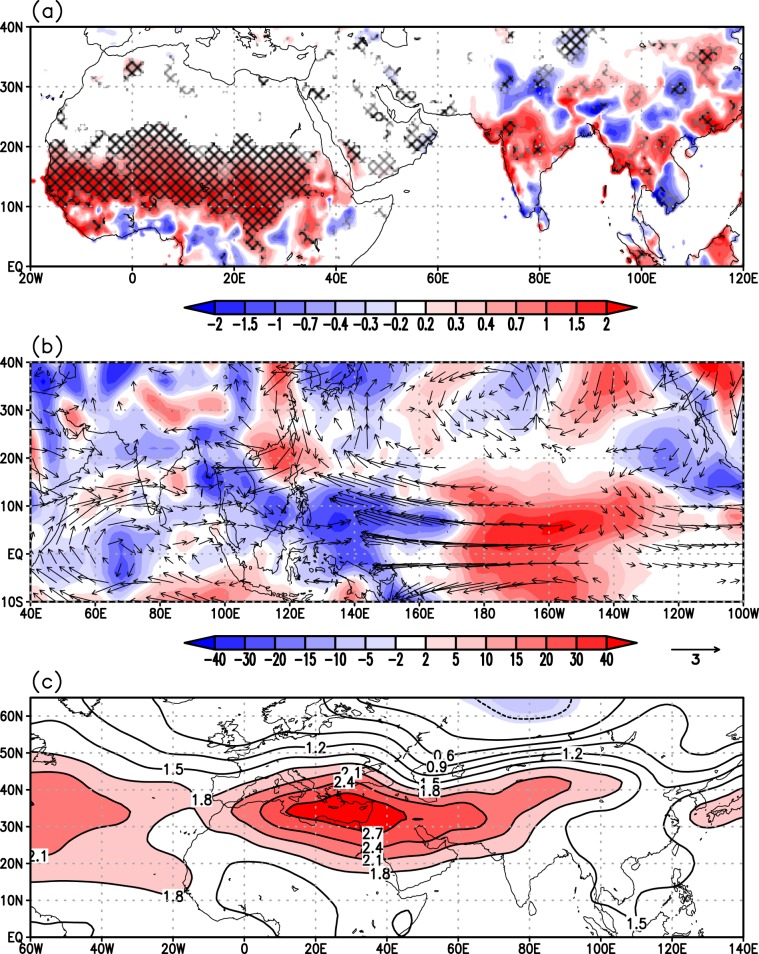
Regressions of observed low-pass filtered June-September (JJAS) mean (**a**) precipitation (mm day-1), (**b**) 500-hPa vertical velocity (hPa day-1) and 850-hPa winds (m s-1), where blue and red areas represent ascending and descending air masses respectively, (**c**) mean upper-tropospheric (200 – 500 hPa) air temperature (°C) on the AMO index shown in Fig. 6 during 1901-2012. All data were linearly detrended prior to the analysis. Precipitation data are from the CRU-TS3.21 dataset, while winds and air temperature are from NOAA-CIRES 20^th^ Century Reanalysis dataset. Anomalies are given per °C change of the AMO. The cross-hatching marks regions where the correlation exceeds the 90% (grey) and 95% (black) confidence levels, estimated by using a Monte Carlo approach with 1,000 random samples of the time series.

The AMO has been suggested to have played a major role in modulating hemispheric-scale temperature gradients at multi-decadal time scales during the last 2000 years^40,41^ via its link with Atlantic Thermohaline Circulation (THC) variations and associated inter-hemispheric heat transport fluctuations^42,43^. The AMO has also been suggested to play a major role in modulating the 20^th^ century multi-decadal variations of Indian and African (Sahel) summer rainfall, as well as the tropical Atlantic atmospheric circulation (e.g., refs. ^17,44^). Figure 5 shows regression/correlation patterns of JJAS precipitation (Fig. 5a), three-dimensional circulation (Fig. 5b), and upper-tropospheric temperature anomalies (Fig. 5c) on the AMO. A positive AMO phase is associated with overall increased precipitation over the Indian subcontinent, with a large positive anomaly over central India (peak values statistically significant at the 95% level) and reduced precipitation to the south and over the central Himalayas (Fig. 5a). This pattern is associated with stronger low-tropospheric southwesterly winds over the Arabian Sea bringing more moisture toward India (Fig. 5b). The mid- and upper-tropospheric heating anomaly (Fig. 5c) features significant warming from the Atlantic across northern Africa to the Middle East and northern India, which intensifies the meridional thermal contrast between the continent and the Indian Ocean- a pattern consistent with several other modelling studies^45,46^. The regional circulation anomalies associated with the southwesterlies in the northern Indian Ocean are part of a hemispheric-wide response pattern to AMO variability featuring enhanced easterly trade winds across the northern equatorial Pacific and enhanced Walker cell north of the equator, leading to larger ascent (precipitation) over Indo-china and the Maritime Continent and subsequent changes in the meridional direction (regional Hadley circulation). These patterns, including the Indo-Pacific sea surface temperature anomalies, bear remarkable similarity to those described in previous studies^47^ and related to variations in the Atlantic oceanic circulation. Figure 5b also displays an interesting wave pattern in the ascending and descending air masses in the northern hemisphere extratropics which some have indicated as responsible for the propagation of the AMO signal from the Atlantic across Eurasia (e.g., ref. ^48^). This analysis, albeit not exhaustive, is suggestive of an AMO-ISM teleconnection with signatures of both an atmospheric pathway via modulation of the upper-tropospheric temperature across Eurasia (e.g.^45,48–50^), and one that also involves atmosphere-ocean feedbacks in the Indo-Pacific region (e.g.^47,51^). Overall, this is indicative of an AMO imprint on the global interhemispheric temperature gradient with a positive phase of the AMO associated with a warmer NH than the SH (Fig. 4b), with ensuing modulation of the NH monsoon systems. We explore this further using palaeoclimate and observational time series.

**Figure 6:**
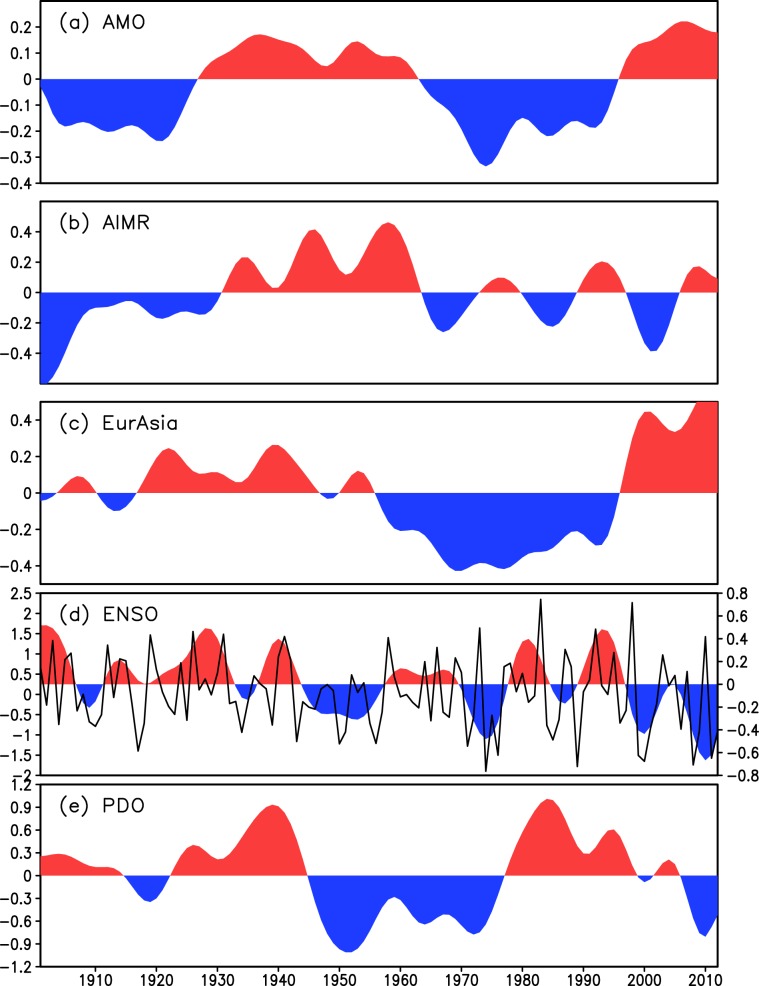
Time series of observed low-pass filtered (**a**) Atlantic Multidecadal Oscillation (AMO) index (°C), (**b**) June-September (JJAS) All-India summer monsoon rainfall anomalies (mm day-1), (**c**) JJAS surface temperature anomalies (°C) over Eurasia (20° - 140°E, 30° - 75°N), (**d**) December-February Niño3.4 index (°C, right y-axis), and (**e**) standardised annual mean Pacific Decadal Oscillation index during 1901–2012. All data were linearly detrended prior to the analysis. The analysis focuses on decadal or longer scale variability, isolated by applying a low-pass Lanczos filter with a cut-off frequency of 10 years to the detrended data. Land temperature data are from the Climate Research Unit (CRU), University of East Anglia, United Kingdom, dataset (CRU-TS3.21) at 0.5° × 0.5° resolution^68^, while sea surface temperature are from the Hadley Centre Sea Ice and Sea Surface Temperature data set (HadISST) dataset version 1 at 0.5° × 0.5° resolution^69^. Rainfall data are taken from the homogeneous rainfall data set of 306 rain gauges in India, developed by the Indian Institute of Tropical Meteorology^70^. The AMO index is defined as the area average over the North Atlantic (80°W - 0°E, 0° - 60°N) of annual mean sea surface temperatures^17^. The Niño3.4 index is calculated by averaging the time series of sea surface temperature (SST) anomalies over (5°S-5°N, 170°-120°W), with SST coming from the HadISST dataset as in Fig. 4. The unfiltered (yearly) values of the Niño3.4 index are also plotted in (**a**) as the continuous black line (left y-axis). The PDO index (obtained from http://research.jisao.washington.edu/pdo/PDO.latest) is derived as the leading principal component of monthly sea surface temperature anomalies in the North Pacific Ocean, poleward of 20°N. The monthly mean global average SST anomalies are removed to separate this pattern of variability from any global warming signal that may be present in the data.

The time series displayed in Fig. 6a–c show that, in general, decadal-scale wet periods over India (e.g., 1926–1965) are in phase with a positive AMO (warmer North Atlantic SSTs) and warmer Asian surface temperature anomalies; conversely, dry periods of the ISM (i.e., 1901–1926 and 1965–1995) occur simultaneously with a negative phase of the AMO and cooler Asian surface temperature anomalies. Such forcing mechanism and distal teleconnections appear to be at play also during the last 2000 years based on our paleo-monsoon reconstructions (Fig. 2) revealing a coherent response between AMO and ISM, further substantiating previous studies based on sparse monsoon records^24,52^. On millennial to century time scales, ISM strength has been correlated with Dansgaard–Oeschger cycles in Greenland ice cores, where weak monsoon events are associated with cold events in the NH during the last glaciation and the Holocene^52–56^. Such weakening of the ISM has been related to Northern hemispheric cooling resulting from ice-sheet instability inducing changes in the Atlantic THC^32,57^. Therefore, a strong relationship between NHT and Indian monsoon variability appears to be a persistent and coherent feature of the past and observed climate at decadal or longer time scales.

In contrast, the association of ISM rainfall with ENSO/Pacific Decadal Oscillation (PDO) is poor in both observational data (Fig. 6d,e) and palaeoclimate records on multi-decadal time scales (Figs. S7 and S8). This is in contrast with one recent study of the last 500 years, which revealed that AMO, ISM rainfall and ENSO exhibit a common 50–80 year variability suggesting that this mode is an integral part of global multi-decadal oscillations arising from large scale coupled ocean-atmosphere-land interactions^58^. Collectively, such discordance suggests that ENSO forcing on ISM rainfall is time varying and complex as suggested by several recent studies^59,60^. This is perhaps because ENSO forcing mechanism operates mainly on higher frequencies on which internal dynamics of monsoon itself dominates and hence not always detected in longer time scales. High frequency variability in rainfall associated with the ENSO^4^, the Indian Ocean Dipole^5^ and Tibetan snow cover^6^ are relatively small, the interannual standard deviation of summer-mean rainfall being around 10% of the climatological mean^10^. Observational data also suggest that the decadal pattern of ocean-atmospheric oscillations of the Pacific Ocean do not conform with the typical ENSO or PDO modes (e.g., Figs. 4b and 5b). Further investigations are required to understand fully the nature of this oscillation pattern.

In summary, we suggest that multi-decadal ISM rainfall variability during the last 2000 years was modulated by AMO related fluctuations in NH temperatures whereby warmer (cooler) Asian landmasses along with a cooler (warmer) Indian Ocean set up stronger (weaker) surface and tropospheric meridional temperature gradients strengthening (weakening) monsoon circulation and rainfall. Therefore, the reconstructed temporal relationships between ISM rainfall, BoB SST and NHT over the last 2000 years allow to extend and strengthen the findings based on the rather limited observational record for the 20^th^ century shedding light on distal teleconnections underpinning multi-decadal ISM variations. It is interesting to note that Fig. 6 suggests a weakening of the AMO-ISMR relationship from the mid-1990s, when the AMO entered a positive phase while the ISM rainfall remained below average (ref. ^17^ Fig. 6). This recent decoupling is discussed further in the context of anthropogenic forcing.

## Implications for ISM Variations Under Anthropogenic Forcing

Our results suggesting a link between the AMO and the associated NH differential warming and ISM rainfall variability at multi-decadal timescales during the last 2 millennia have important implications for the current debate on the future evolution of ISM rainfall in response to global warming. Our study strongly supports the notion that multi-decadal variations of ISMR are sensitive to extra tropical forcing that occurs beyond regional scales. It is also on these timescales that anthropogenic forcing can interact with internal variability of the monsoon. Broadly, AMO forcing on the monsoon acts by changing inter-hemispheric thermal gradients (Fig. 4). NH warming associated with positive phases of this oscillation enhances meridional pressure gradients that drive cross-equatorial flows and intensify NH monsoon circulation and rainfall^17^ (Fig. 5). Global warming trends since the early seventies have been asymmetric across the hemispheres, with the NH warming faster than the SH^23,61^. The interaction between this global warming pattern and positive phases of the AMO would further accentuate the inter-hemispheric thermal contrast, reinforcing NH temperature positive anomalies and intensifying NH monsoon circulation and rainfall. The post mid-1990s decoupling of ISM from AMO (Fig. 6) has been widely attributed to the influence of aerosols and land use changes^8,11,19^. In view of our results, such regional influences dampening ISMR are likely to be temporary depending partly on future levels of these aerosol emission and land use change. On longer time scales, NH temperature anomalies will have an overriding influence on enhancing monsoon. In this regard, we note that ISM rainfall registered a recovery since 2002 with above average rainfall after 2012^2,23,43,62^. Finally, we suggest that interaction between AMO and global warming is likely to be a crucial factor in ISM rainfall trajectory into the future. Therefore, large-scale interactions involving extratropical factors in response greenhouse gas warming cannot be ignored in future ISM rainfall prediction.

## Methods Summary

Core MD161/17 was collected at a water depth of 790 m from the KGB. Age model was based on linear interpolation between 11 AMS radiocarbon dates which covers a time span of 2 kyr (Supplementary Table 2). *Globigerinoides ruber* (150 to 250 µm) was analyzed for δ^18^O by reacting with 100% orthoposphoric acid at a constant 75 °C in a Kiel Carbonate III preparation device and evolved CO_2_ gas was analyzed by using Thermo Electron delta plus advantage stable isotope ratio mass spectrometer. Cleaning protocol of *G. ruber* for Mg/Ca analyses were followed by Barker *et al*.^63^, in addition a reductive cleaning step was used according to Martin and Lea^64^. Mg/Ca ratios were measured by using an axial viewing Varian 720 ICP-OES (for details see the supplementary material). Mg/Ca values were then used to estimate SSTs using the equation Mg/Ca = 0.449exp(0.09*T)^65^. δ^18^Osw were computed by applying the following equation of Bemis *et al*.^66^: δ^18^Osw = 0.27 ((T − 16.5 + 4.8*d18O)/4.8). The derived δ^18^Osw estimates were corrected for continental ice volume using Shackleton’s data set^67^ and presented here as δ^18^Ow. SST and δ^18^Ow anomalies are computed by subtracting the mean values of all data points within two millennia from the SST and δ^18^Ow values respectively.

## Supplementary information

Supplementary file.
