# Supplementary material for: Coherent response of the Indian Monsoon Rainfall to Atlantic Multi-decadal Variability over the last 2000 years
Source: Sci Rep. 2020 Jan 28;10:1302. doi: 10.1038/s41598-020-58265-3 (PMC6987308; doi:10.1038/s41598-020-58265-3)
Supplement: Supplementary file 1 — Supplementary file. [file 41598_2020_58265_MOESM1_ESM.pdf]

Supplementary Information

Coherent response of the Indian Monsoon Rainfall to Atlantic Multi-decadal Variability over the last 2000 years

Pothuri Divakar Naidu*[1]., Raja Ganeshram[2]., Massimo Bolasina[2]., Champoungam Panmei[1,3]., Dirk Nürnberg[4]., Jonathan F. Donges[5,6]

1. CSIR-National Institute of Oceanography, Dona Paula 403004, Goa, India

2. School of Geosciences, University of Edinburgh, Edinburgh, UK

3. Academy of Scientific and Innovative Research (AcSIR), CSIR-NIO, Goa, India

4.GEOMAR, Wischhofstrasse 1-3, 24148 Kiel, Germany

5. Postdam Institute for Climate Impact Research, P.O. Box 601203, D-14412 Postdam, Germany

6. Planetary Boundary Research Lab, Stockholm Resilience Center, Stockholm University, Sweden

1. Processing of sediment samples and Establishment of chronology

Core MD161/17 was sampled at 1 cm interval up to 11m core depth. Sediment samples were disaggregated using distilled water and then wet sieved in >150 µm sieve. Mixed planktonic foraminifera from the >150µm size fraction were picked and used for radiocarbon dating. Radiocarbon measurements were performed at the University of Arizona, USA. Radiocarbon ages were converted to calendar ages using the Calib 7.1[1] and the Marine13 calibration curve[2] and listed details of age control points in Table 1. Available reservoir estimates for the Bay of Bengal surface waters are not substantially different than the standard marine reservoir correction[3,4], which we used to calibrate our data. Calibrated ages versus depth are shown in supplementary figure 1. Ages for samples between calibrated dates were obtained by linear interpolation.

2. Oxygen isotopes analyses of Planktonic foraminifera

6 to 8 tests of *Globigerinoides ruber* (white) from the 150-250 μm size fraction were analyzed for stable oxygen ($\delta^{18}O$) and carbon isotopes ($\delta^{13}C$). 0.1 mg of carbonate material were reacted with 100% orthophosphoric acid at 75°C in a Kiel Carbonate III preparation device and the resulting CO_2 was then analysed on a Thermo Electron Delta Plus Advantage stable isotope ratio mass spectrometer, University of Edinburgh, UK. The standard deviation (n = 163) of a powdered coral laboratory standard (COR1D, $\delta^{13}C$ = -0.648, $\delta^{18}O$ = -4.924) run along with samples included in this study was ± 0.05‰ for $\delta^{13}C$ and ± 0.06‰ for $\delta^{18}O$. All carbonate isotopic values are reported to relative to the Vienna Pee Dee Belemnite (VPDB) standard and presented in supplementary figure 2.

3. Magnesium/Calcium (Mg/Ca) Analyses

30-40 tests of *G. ruber* (white) from the 150-250 μm size fraction were used for Mg/Ca analyses. The sample preparation for Mg/Ca measurements in foraminiferal calcite followed the protocol of Barker et al.[5] but included a reductive cleaning step according to Martin and Lea[6]. Mg/Ca analyses were performed on an axial viewing Varian 720 ICP-OES at GEOMAR. Prior to measurements, dilution of sample solutions with yttrium water (concentration 112.5 μmol/l) was performed to detect possible drifts of the ICP-OES during the analyses. The Mg/Ca measurements were drift corrected and standardized to the internal ECRM 752-1 consistency standard (3.761 mmol/mol Mg/Ca; Greaves et al.[7]). The external reproducibility for the ECRM standard for Mg/Ca is ±0.1 mmol/mol (2**σ** s.d), replicate measurements run during different sessions exhibit a reproducibility of maximum ~0.2 mmol/mol (2**σ** s.d). All samples were monitored for their Fe/Ca, Al/Ca and Mn/Ca ratios to avoid post-depositional contamination[8]. For most of the samples, Mn/Ca ratios were below the detection limit. Samples with Al/Ca and Fe/Ca are higher than the (regional) threshold of 0.1 mmol/mol (Barker et al.[5]) were excluded or reanalysed after further cleaning. Overall, the Fe/Ca, Al/Ca and Mn/Ca ratios do not correlate with the Mg/Ca ratios. We therefore consider our Mg/Ca data is not affected by sample contamination. Mg/Ca values were then used to estimate SSTs using the equation Mg/Ca = 0.449exp(0.09*T) (Anand et al.[9]), where Mg/Ca is in mmol/mol and SST is in °C shown in supplementary figure 2. $\delta^{18}Osw$ were computed by applying the following equation of Bemis et al.[10]: $\delta^{18}Osw = 0.27 ((T - 16.5 + 4.8*\delta^{18}O)/4.8)$. The derived $\delta^{18}Osw$ estimates were corrected

for continental ice volume using Shackleton[11] data set and presented here as δ^{18}Ow in supplementary figure 2. The foraminifera lysocline lies around 2600m in this region[12], the location of the core well above the lysocline depth, therefore carbonate dissolution effect on the Mg/Ca are expected to be very small. The errors in δ^{18}Ow and SST reconstructions are estimated by propagating error introduced by δ^{18}O, Mg/Ca measurements, and the Mg/Ca:calibration and temperature equations. The resulting error estimates are an average of 1°C for SST and 0.3‰ for δ^{18}Ow. We refrain from converting the δ^{18}Ow values into salinity values as it is not warranted that the modern linear relationship between δ^{18}Ow and salinity held through time.

4. Geography and Climatology

The Indian peninsula is bordered on the west by the Arabian Sea and on the east by the Bay of Bengal, in the south by Indian Ocean and in the north by Tibetan plateau. Important geographic features are the Thar dessert to the northeast, the Indo-Gangetic Plain to the south of the Himalayas, which lies between the Indus and Ganga (Ganges) rivers, and the Deccan Plateau. Western Ghats is a mountain range that runs parallel to the western Coast of the Indian Peninsula. A discontinuous range of mountains runs parallel to the Bay of Bengal Coast known as Eastern Ghats, which have been eroded by the four major peninsula rivers of Godavari, Krishna, Mahanadi and Kaveri. The Godavari Basin covers an area of 312,812 km^2, representing about 12% of the area of continental India[13].

Indian monsoon plays an important role on the climatology of the Indian Peninsula with strong contrast of wet and dry seasons associated with seasonal reversal of monsoon winds in the Indian Ocean. During June through September precipitation is brought in by the moist southwest winds which accounts about 85% of the rain fall in India and also overhead precipitation in the Bay of Bengal. Prevailed strong coupling between rainfall and river discharge into the Bay of Bengal results freshening of surface waters in the coastal regions, especially near the mouths of major rivers (Ganges-Bramhaputra, Godavari, Krishna, Mahanadi). It is evident from Modern salinity patterns that the strongest salinity variations in the western Bay of Bengal occur in front of the Krishna and Godavari mouths[14] and during the Holocene, the Godavari delivered ample sediment quantities to the continental slope, giving rise to an expanded sedimentary sequence[15]. For that reason we have collected the Core MD161/17 from the Krishna-Godavari Basin in order

to trace the multi-decadal variability of Indian Summer Monsoon (ISM) rainfall in the catchment regions of the Krishna-Godavari in the Deccan peninsula. As salinity fluctuations occur simultaneously, with the appearance of a coastal freshwater plume fed by Indian rivers during the ISM season, we have used δ^{18}Ow a proxy dependent on the salinity to reconstruct the Indian monsoon rainfall in this study.

Sediment trap studies from the Bay of Bengal have shown that *G. ruber* occurs throughout the year and with peak abundance during SW monsoon[16,17]. *G. ruber* is abundant in the surface mixed layer and (~100m) but its shell growth has been found to be restricted to the top 35 m of water[18]. Hence, Mg/Ca in *G. ruber* provides SST and δ^{18}Ow derived from δ^{18}Oc of *G. ruber* represent Evaporation - Precipitation associated with the SW monsoon. Paired measurements of *G. ruber* for δ^{18}O and Mg/Ca were successfully used to reconstruct SST and SSS previously from the Bay of Bengal[19,20].

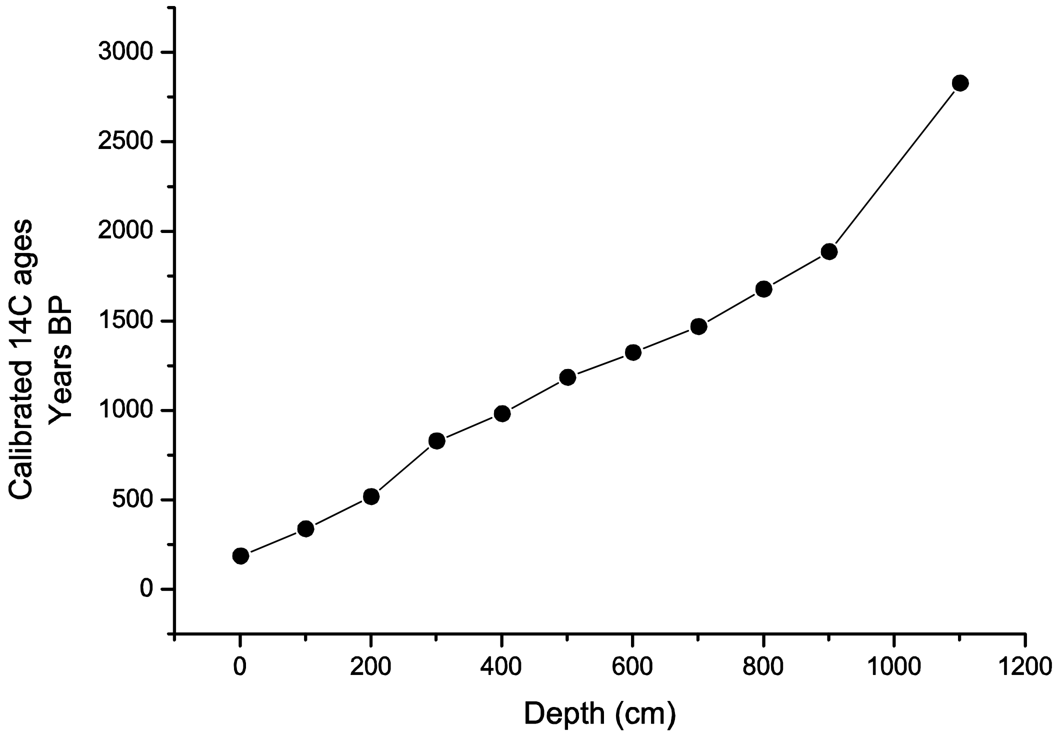

Supplementary Figure 1. Calibrated ^{14}C AMS dates versus depth for the core MD161/17.

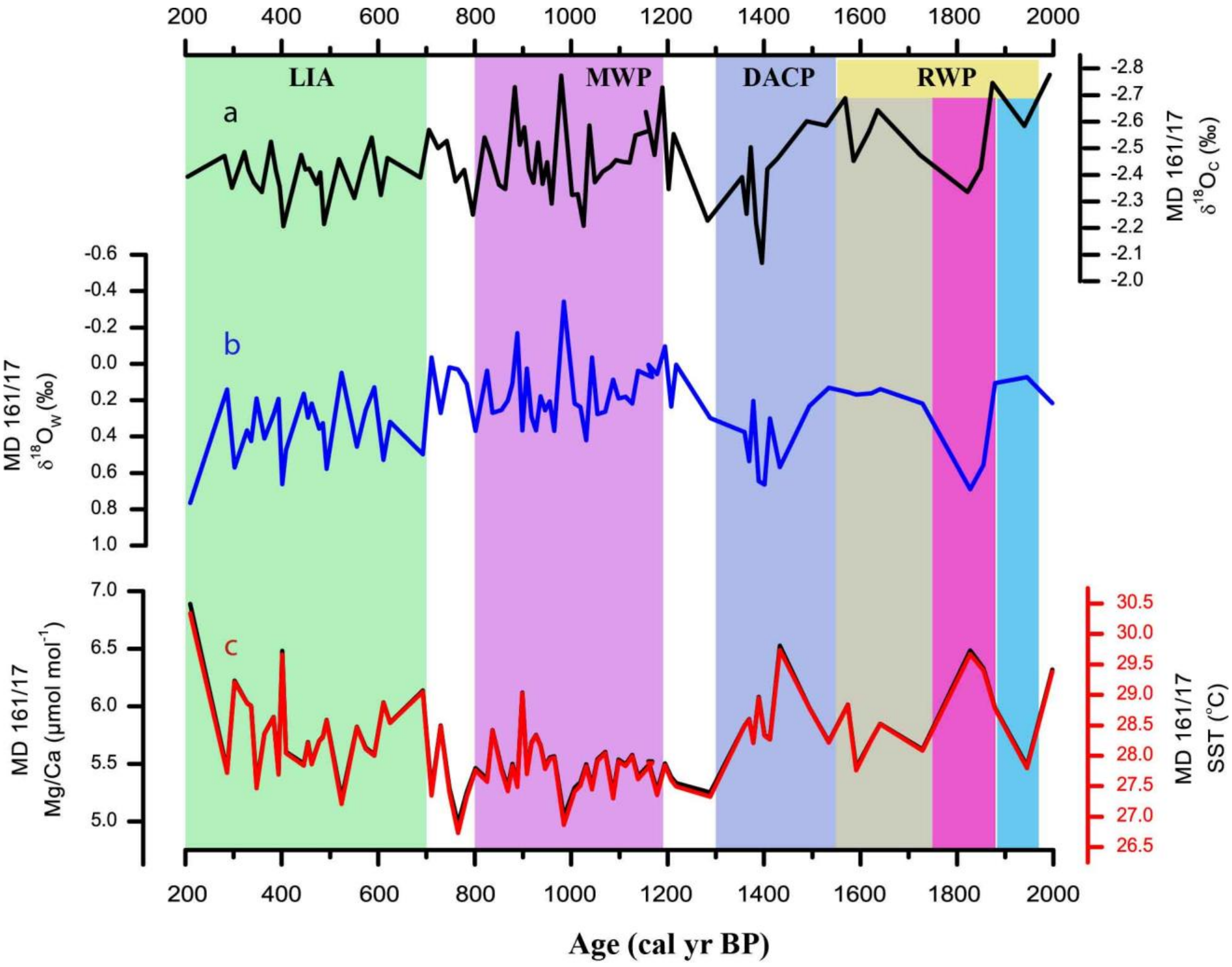

Supplementary Figure 2. Reconstructed δ^{18}Oc and δ^{18}Ow and Mg/Ca derived SST from the Core MD161/17 from the Krishna Godavari Basin representing the ISM rainfall variability over 2000 years BP.

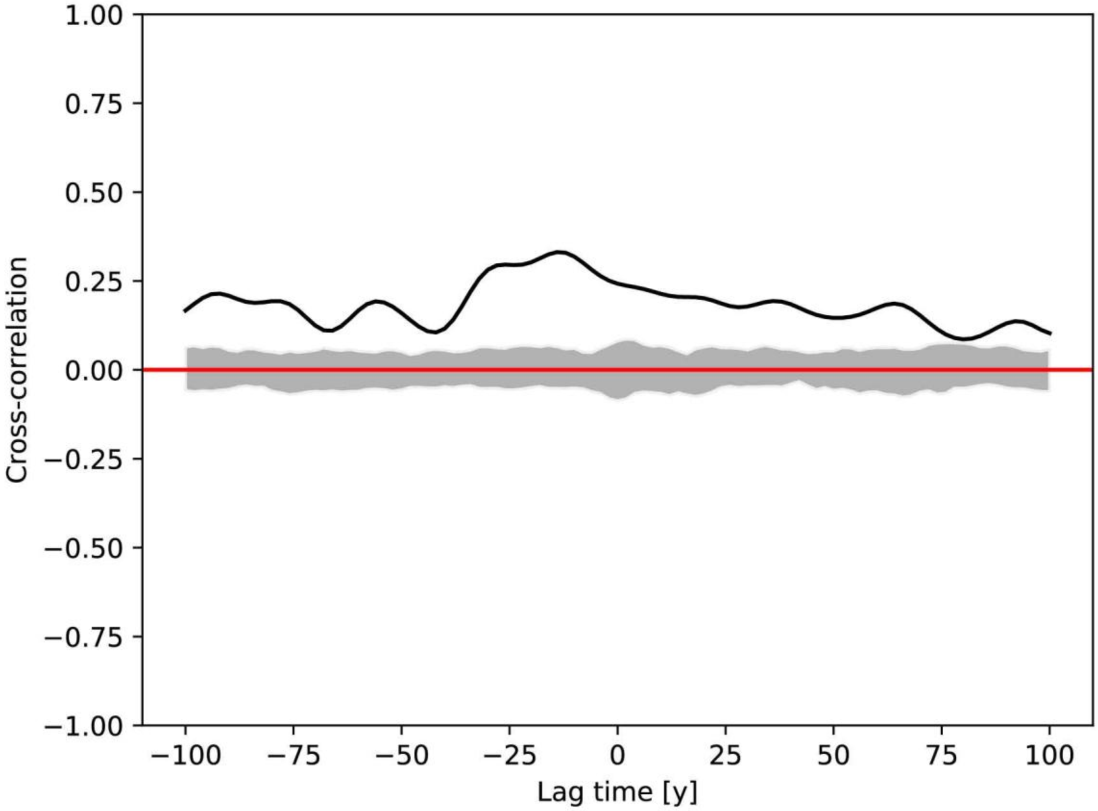

Supplementary Figure 3. Kernel cross-correlation (red line) analysis of δ^{18}Ow (proxy of ISM rainfall) and δ^{18}O of speleothem from Sahiya cave[21]. Note significant positive correlation at zero year lag between the ISM rainfall derived from marine record (MD161/17) and terrestrial record (Sahiya cave). The 90 % confidence band (turqoise) is derived from a test for statistical significance based on uncorrelated time series with the same amplitude distribution as the original data series (obtained by random shuffling[22,23])

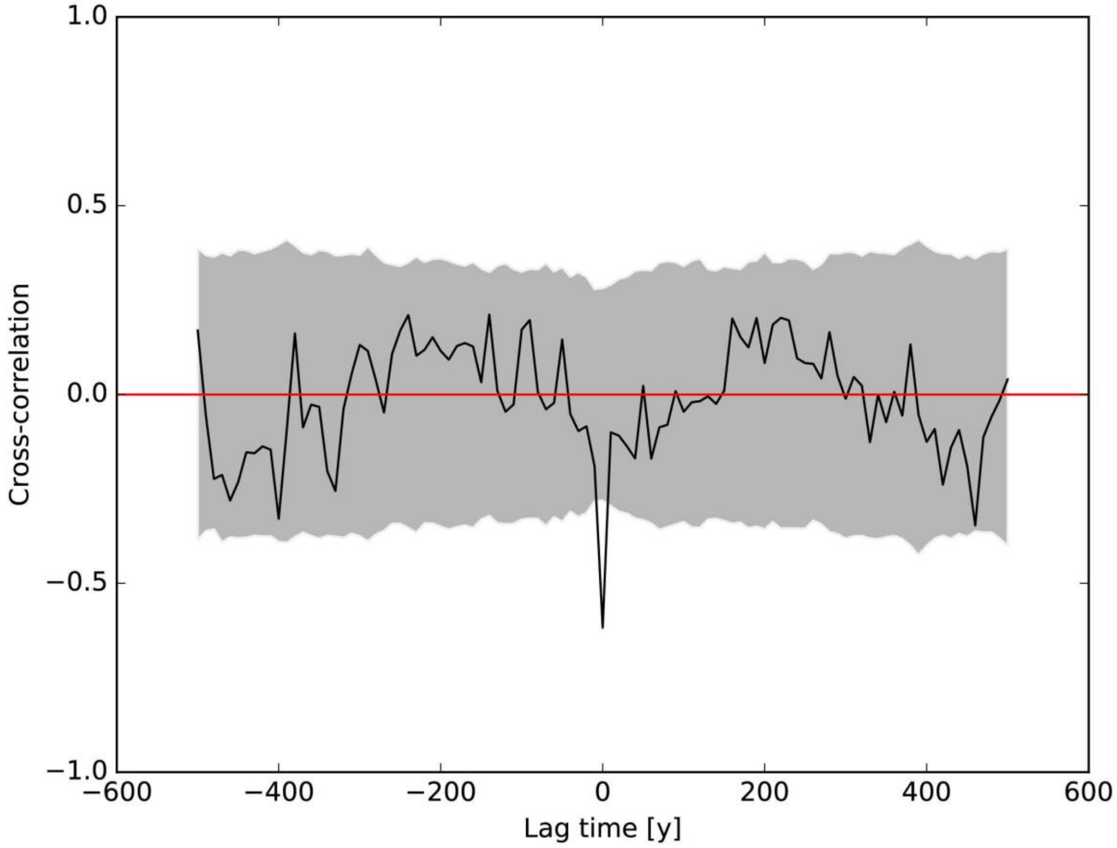

Supplementary Figure 4. Kernel cross-correlation (red line) analysis of δ^{18}Ow (proxy of ISM rainfall) and sea surface temperatures (SST) derived from Mg/Ca ratios in planktonic foraminifera *G. ruber*. Note significant negative correlation at zero year lag between the ISM rainfall and SST in KGB. The 90 % confidence band (turqoise) is derived from a test for statistical significance based on uncorrelated time series with the same amplitude distribution as the original data series (obtained by random shuffling[22,23]).

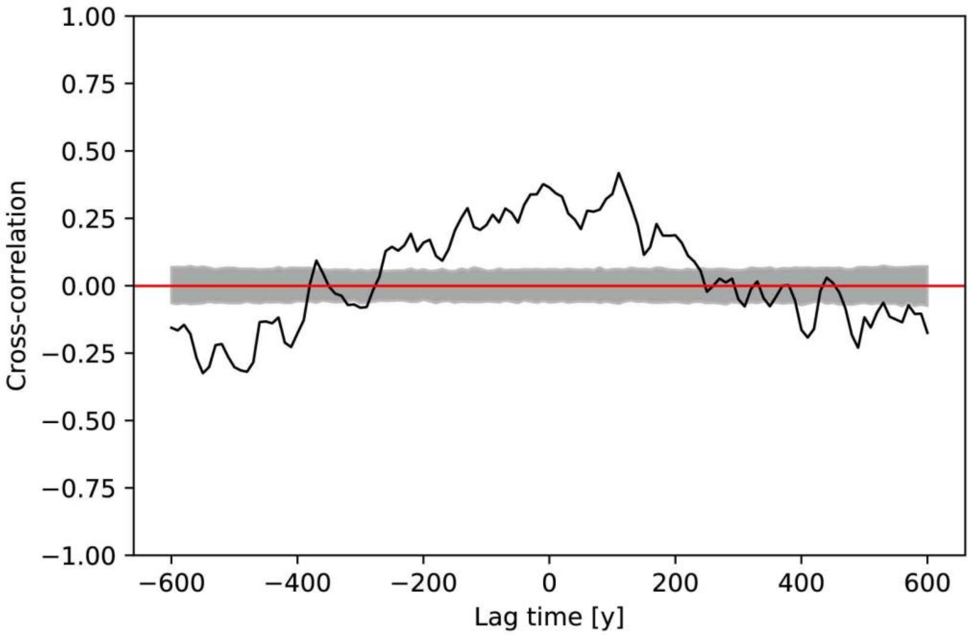

Supplementary Figure 5. Kernel cross-correlation (red line) analysis of δ^{18}Ow anomaly (proxy of ISM rainfall) and Northern Hemisphere Temperature anomaly (NHT). Note significant positive correlation at zero year lag between the ISM rainfall and NHT. The 90 % confidence band (gray) is derived from a test for statistical significance based on uncorrelated time series with the same amplitude distribution as the original data series (obtained by random shuffling[22,23]).

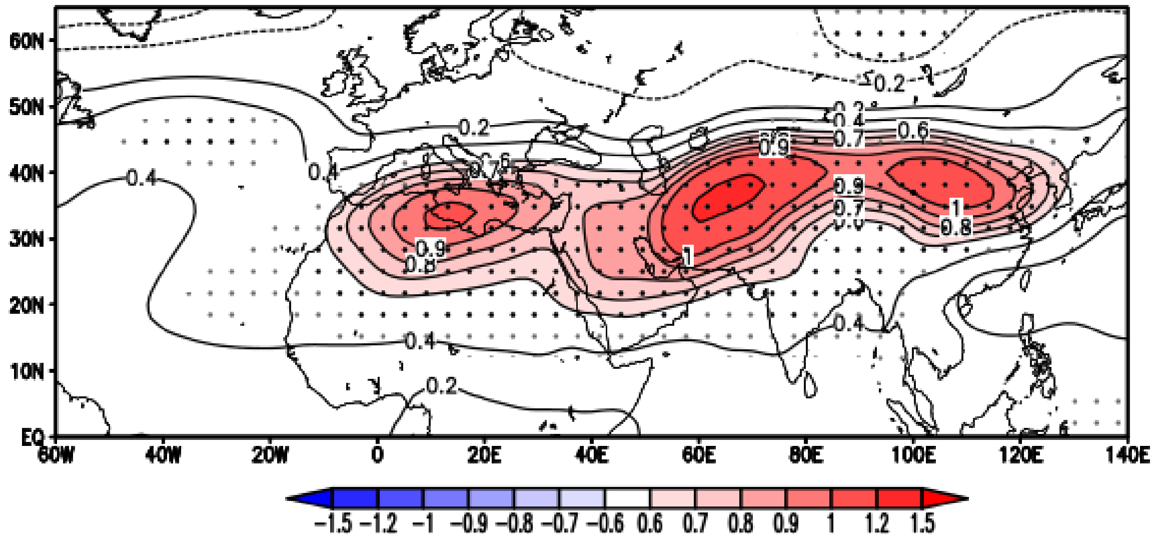
60N
50N
40N
30N
20N
10N
EQ
60W
40W
20W
0
20E
40E
60E
80E
100E
120E
140E
−1.5 −1.2 −1 −0.9 −0.8 −0.7 −0.6 0.6 0.7 0.8 0.9 1 1.2 1.5

Figure 6. Regression of observed low-pass filtered June-September (JJAS) mean upper-tropospheric (200 – 500 hPa) air temperature (°C black contours; anomalies of magnitude larger than 0.6°C are shaded) on observed JJAS All-India summer monsoon rainfall during 1901-2012. All data were linearly detrended prior to the analysis. The analysis focuses on decadal or longer scale variability, isolated by applying a low-pass Lanczos filter with a cut-off frequency of 10 years to JJAS-mean data. The dots mark regions where the correlation exceeds the 90% (grey) and 95% (black) confidence levels, estimated by using a Monte Carlo approach with 1,000 random samples of the time series[24]. Air temperature data are from the NOAA-CIRES 20th Century Reanalysis dataset (V2c) at 2° x 2° resolution[25]. Rainfall data are taken from the homogeneous rainfall data set of 306 raingauges in India, developed by the Indian Institute of Tropical Meteorology[26].

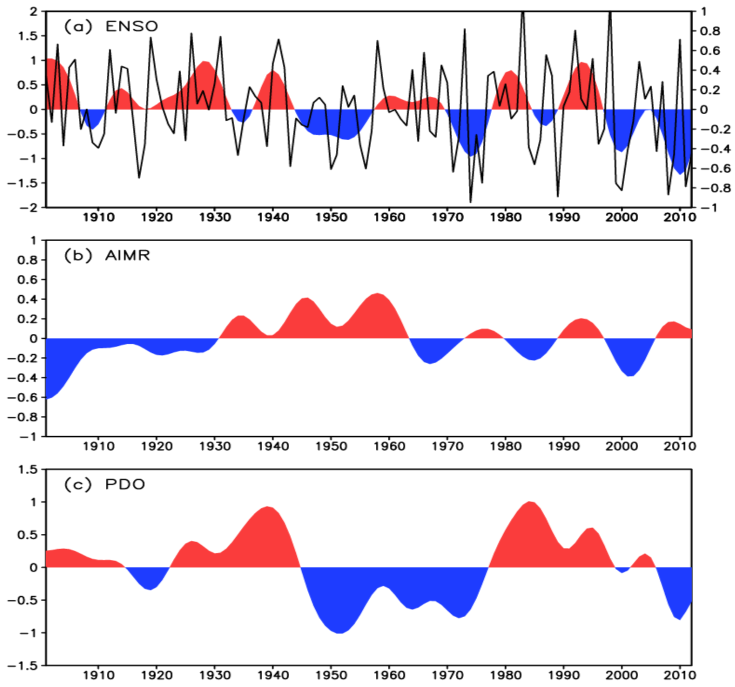

Supplementary Figure 7. Time series of observed (a) December-February Niño3.4 index (°C, right y-axis), (b) June-September (JJAS) All-India summer monsoon rainfall anomalies (mm day^{-1}), and (c) standardised annual mean Pacific Decadal Oscillation index during 1901-2012. All data were linearly detrended prior to the analysis. The analysis focuses on decadal or longer scale variability, isolated by applying a low-pass Lanczos filter with a cut-off frequency of 10 years to the detrended data. The Niño3.4 index is calculated by averaging the time series of sea surface temperature (SST) anomalies over (5°S-5°N, 170°-120°W), with SST coming from the HadISST dataset as in Fig. 6. The unfiltered (yearly) values of the Niño3.4 index are also plotted in (a) as the continuous black line (left y-axis). The monsoon rainfall time series is as in Fig. 6. The PDO index (obtained from http://research.jisao.washington.edu/pdo/PDO.latest) is derived as the leading principal component of monthly sea surface temperature anomalies in the North Pacific Ocean, poleward of 20°N. The monthly mean global average SST anomalies are removed to separate this pattern of variability from any global warming signal that may be present in the data[27].

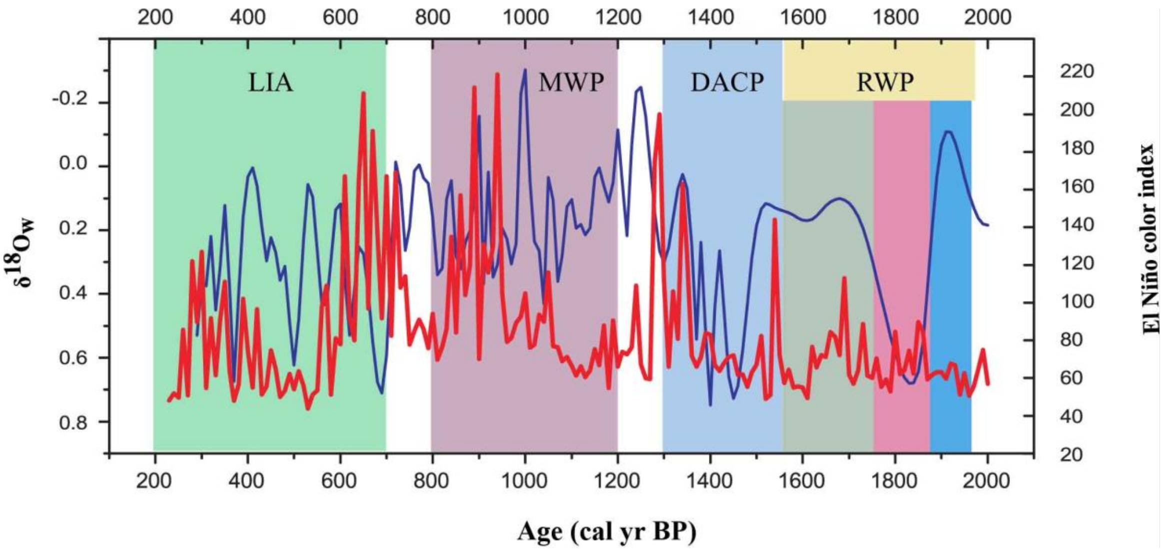

Supplementary Figure 8. ENSO variability reconstructed based on the colour index in a sediment core from Laguna Pallacacocha, Southern Ecuador, which is strongly influenced by ENSO events (red line)[28] and monsoon reconstruction based on $\delta^{18}Ow$ (blue line).

Table 1. AMS ^{14}C dates of sediment intervals dated in the core MD 161/17 and calibrated calendar ages (yr BP), using Calib 7.1[1] and the Marine13 calibration curve[2] with a correction for reservoir effect of 400 years[4].

Serial No.	Core depth (cm)	^{14}C Radiocarbon age (yr BP)	Error	Calibrated Age (calendar yr BP)
1	001	537	±40	186
2	101	749	±30	338.5
3	201	913	±41	517
4	301	1278	±31	828.5
5	401	1439	±34	981.5
6	501	1612	±33	1184.5
7	601	1788	±33	1323.5
8	701	1931	±49	1468.5
9	801	2104	±40	1677.5
10	901	2279	±43	1886
11	1101	3070	±41	2827.5

Table 2. Correlation, partial correlations, rank correlations and partial rank correlations between various proxies.

	Correlation	***Partial Correlation***	***Rank Correlation***	***Partial Rank Correlations***
SST - δ^{18}Ow	r=-0.745 $p<0.001$	r =-0.768 $p<0.001$	r =-0.518 $p<0.001$	r=-0.528 $p<0.001$

References

1. Stuiver, M. & Reimer, P. J. Extended C-14 data-base and revised calib 3.0 C-14 age calibration program. *Radiocarbon* 35(1), 215-230 (1993).

2. Reimer, P. J., *et al*. IntCal and Marine13 radiocarbon age calibration curves 0-50,000 cal years BP. *Radiocarbon* 55(4):1869–1887 (2013).

3. Datta, K., Bhushan, R. & Somayajulu, B. L. K. Delta R correction values for the northern Indian Ocean. *Radiocarbon* 43, 483-488 (2001).

4. Southon, J., Kashgarian, M., Fontugne, M., Metivier, B. & Yim W. W. S. Marine reservoir corrections for the Indian Ocean and southeast Asia. *Radiocarbon* 44(1), 167-180 (2002).

5. Barker, S., Greaves, M. & Elderfield, H. A study of cleaning procedures used for foraminiferal Mg/Ca paleothermometry. *Geochem. Geophys. Geosyst.* 4(9), 8407 (2003).

6. Martin, P. A., & Lea, D. W. A simple evaluation of cleaning procedures on fossil benthic foraminiferal Mg/Ca. *Geochem. Geophys. Geosyst*. 3(10), 8401 (2002).

7. Greaves, M., *et al*. Inter laboratory comparison study of calibration standards for foraminiferal Mg/Ca thermometry. *Geochem. Geophys. Geosyst.* 9, Q08010 (2008).

8. Rosenthall, Y., Field, F., & Sherrell, R. M. Precise determination of element/calcium ratios in calcareous samples using Sector Field inductively coupled plasma mass spectrometry. *Anal. Chem.* 71, 3248-3253 (1999).

9. Anand, P., Elderfield, H., & Conte, M. H. Calibration of Mg/Ca thermometry in planktonic foraminifera from a sediment trap series. *Paleoceanography* 18 (2), 1050 (2003).

10. Bemis, B. E., Spero, H. J., Bijma, J. & Lea, D. W. Re-evaluation of the oxygen isotopic composition of planktonic foraminifera: Experimental results and revised paleotemperature equations. *Paleoceanography* 13, 150–160 (1998).

11. Shackleton, N. J. The 100,000-year ice age cycle identified and found to lag temperature, carbon dioxide, and orbital eccentricity. *Science* 289, 1897–1902 (2000).

12. Cullen, J. L. & Prell, W. L. Planktonic foraminifera of the northern Indian Ocean: Distribution and preservation in surface sediments. *Mar. Micropaleontol.* 9: l-52 (1984).

13. Ponton, C., Giosan, L., Eglinton, T. I., Fuller, D. Q., Johnson, J. E., Kumar, P., & Collett, T. S. Holocene aridification of India. *Geophys. Res. Lett.* 39, L03704 (2012).

14. Antonov, J. I., Locarnini, R. A., Boyer, T. P., Mishonov, A. V. & Garcia H. E. Sea Surface Salinity: In *World Ocean Atlas 2005, Vol. 2: Salinity,* edited by S. Levitus, Ed., NOAA, Washington, D.C. (2006).

15. Forsberg, C. F., Solheim, A., Kvalstad, T. J., Vaidya, R. & Mohanty S. Slope instability and mass transport deposits on the Godavari river delta, east Indian margin from a regional geological perspective. *Submarine Mass Movements and Their Consequences 27*, 19-27 (2007).

16. Gupta, M. V. S., Curry, W. B., Ittekkot, V., & Muralinath, A. S. Seasonal variation in the flux of planktonic foraminifera; sediments trap results from the Bay of Bengal, Northern Indian Ocean. *J. Foraminiferal Res*. 27, 5-19 (1997).

17. Unger, D., Ittekkot,V., Schafer, P., Tiemann, J. & Reschke, S. Seasonality and interannual variability of particle fluxes to the deep Bay of Bengal: influence of riverine input and oceanographic processes. *Deep-Sea Res. Part 2 Top. Stud. Oceanogr 50*(5), 897-923 (2003).

18. Fairbanks, R. G., Sverdlove, M., Free, R., Wiebe, P. H. & Be A. W. H. Vertical distribution and isotopic fractionation of living planktonic-foraminifera from the Panama Basin. *Nature* 298(5877), 841-844 (1982).

19. Rashid, H., Flower, B. P., Poore, R. Z., Quinn, T. M. Indian Ocean monsoon variability record from the Andaman Sea. *Quat. Sci. Rev.* 26, 2586–2597 (2007).

20. Govil, P., & Naidu, P. D.Variations of Indian monsoon precipitation during the last 32 kyr reflected in the surface hydrography of the Western Bay of Bengal. *Quat. Sci. Rev.* 30, 3871-3879 (2011).

21. Kathayat, G., Cheng, H., Sinha, A., Yi, L., Li, X., Zhang, H., Li, H., Ning, Y. & Edwards, R. L. The Indian monsoon variability and civilization changes in the Indian subcontinent. *Sci. Adv.* 3:e1701296 (2017).

22. Rehfeld, K., Marwan, N., Heitzig, J., & Kurths, J. Comparison of correlation analysis techniques for irregularly sampled time series. *Nonlin. Processes Geophys.* 18(3), 389-404 (2011).

23. Donges, J. F., Donner, R., Marwan, N., Breitenbach, S. F., Rehfeld, K., & Kurths, J. Non-linear regime shifts in Holocene Asian monsoon variability: potential impacts on cultural change and migratory patterns. *Clim. Past.* 11(5), 709-741 (2015).

24. McCabe, G. J, Palecki, M. A., & Betancourt, J. L. Pacific and Atlantic Ocean influences on multidecadal drought frequency in the United States. *Proc. Natl. Acad. Sci.* 101: 4136–4141 (2004).

25. Compo, G. P., Whitaker, J. S., Sardeshmukh, P. D., Matsui, N., Allan, R. J., Yin, X., Gleason, B. E., Vose, R. S., Rutledge, G., Bessemoulin, P., Brönnimann, S., Brunet, M., Crouthamel, R. I., Grant, A. N., Groisman, P. Y., Jones, P. D., Kruk, M., A. C. Kruger, A. C., Marshall, G. J., Maugeri, M., Mok, H. Y., Nordli, Ø., Ross, T. F., Trigo, R.M., Wang, X. L., Woodruff, S. D. & Worley, S. J.: The twentieth century reanalysis project. *Quarterly J. Roy. Meteorol. Soc.* 137, 1-28 (2011). doi: 10.1002/qj.776.

26. Kothwale, D. R. & Rajeevan, M. Monthly, seasonal and annual rainfall time series for All-India. Homogenous regions and meteorologyical subdivisions: 1871-2016 (2017). Research Report No. RR138.

27. Mantua, N. J., Hare, S. R., Zhang, Y., Wallace, J. M. & Francis, R. C: A Pacific interdecadal climate oscillation with impacts on salmon production. *Bull. Amer. Meteor. Soc.* 78, 1069-1079 (1997).

28. Moy, C. M., Seltzer, G. O., Rodbell, D. T. & Anderson, D. M. Variability of El Niño/Southern Oscillation activity at millennial time-scales during the Holocene epoch. *Nature* 420, 162–165 (2002).
